# Analysis of the advantage of individual PTVs defined on axial 3D CT and 4D CT images for liver cancer

**DOI:** 10.1120/jacmp.v13i6.4017

**Published:** 2012-11-08

**Authors:** Fengxiang Li, Jianbin Li, Jun Xing, Yingjie Zhang, Tingyong Fan, Min Xu, Dongping Shang, Tonghai Liu, Jinlong Song

**Affiliations:** ^1^ Department of Radiation Oncology Shandong Cancer Hospital and Institute Jinan China; ^2^ Big bore CT Room Shandong Cancer Hospital and Institute Jinan China; ^3^ Radiation Physics Shandong Cancer Hospital and Institute Jinan China; ^4^ Hepatobiliary Interventional Radiology Shandong Cancer Hospital and Institute Jinan China

**Keywords:** liver tumor, radiotherapy, 4D CT, planning target volumes, target volume comparison

## Abstract

The purpose of this study was to compare positional and volumetric differences of planning target volumes (PTVs) defined on axial three dimensional CT (3D CT) and four dimensional CT (4D CT) for liver cancer. Fourteen patients with liver cancer underwent 3D CT and 4D CT simulation scans during free breathing. The tumor motion was measured by 4D CT. Three internal target volumes (ITVs) were produced based on the clinical target volume from 3DCT (${\text{CTV}}_{3D}$): i) A conventional ITV (${\text{ITV}}_{\text{conv}}$) was produced by adding 10 mm in CC direction and 5 mm in LR and and AP directions to ${\text{CTV}}_{3D}$; ii) A specific ITV (${\text{ITV}}_{\text{spec}}$) was created using a specific margin in transaxial direction; iii) ${\text{ITV}}_{\text{vector}}$ was produced by adding an isotropic margin derived from the individual tumor motion vector. ${\text{ITV}}_{4D}$ was defined on the fusion of CTVs on all phases of 4D CT. PTVs were generated by adding a 5 mm setup margin to ITVs. The average centroid shifts between PTVs derived from 3DCT and ${\text{PTV}}_{4D}$ in left–right (LR), anterior–posterior (AP), and cranial–caudal (CC) directions were close to zero. Comparing ${\text{PTV}}_{4D}$ to ${\text{PTV}}_{\text{conv}}$, ${\text{PTV}}_{\text{spec}}$, and ${\text{PTV}}_{\text{vector}}$ resulted in a decrease in volume size by 33.18% ±12.39%, 24.95% ±13.01%, 48.08% ±15.32%, respectively. The mean degree of inclusions (DI) of ${\text{PTV}}_{4D}$ in ${\text{PTV}}_{\text{conv}}$, and ${\text{PTV}}_{4D}$ in ${\text{PTV}}_{\text{spec}}$, and ${\text{PTV}}_{4D}$ in ${\text{PTV}}_{\text{vector}}$ was 0.98, 0.97, and 0.99, which showed no significant correlation to tumor motion vector (r=‐0.470, 0.259, and 0.244; p=0.090, 0.371, and 0.401). The mean DIs of ${\text{PTV}}_{\text{conv}}$ in ${\text{PTV}}_{4D}$, ${\text{PTV}}_{\text{spec}}$ in ${\text{PTV}}_{4D}$, and ${\text{PTV}}_{\text{vector}}$ in ${\text{PTV}}_{4D}$ was 0.66, 0.73, and 0.52. The size of individual PTV from 4D CT is significantly less than that of PTVs from 3DCT. The position of targets derived from axial 3DCT images scatters around the center of 4D targets randomly. Compared to conventional PTV, the use of 3D CT‐based PTVs with individual margins cannot significantly reduce normal tissues being unnecessarily irradiated, but may contribute to reducing the risk of missing targets for tumors with large motion.

PACS number: 87

## I. INTRODUCTION

Traditionally, the role of radiotherapy in liver cancer has been limited due to the relative radiosensitivity of the liver.^1^, ^2^ With the use of three‐dimensional radiotherapy (3D CRT) and image‐guided radiotherapy (IGRT), radiotherapy has been accepted for treatment of liver cancer.^1^, ^3^ However, the conventional 3D CRT for liver cancer is generally based on axial three‐dimensional CT (3D CT) scanning. Fast 3D CT images are unable to encompass respiration‐induced liver tumor motion because they could only provide a snapshot of anatomy.^(4)^ So, another empirical margin is expanded to include tumor motion, although such margin may introduce normal tissue being unnecessarily irradiated or a geometric miss.^(5)^

Currently, the technique of 4D CT has been widely used in the radiation therapy of lung cancer. The use of 4D CT scanning is not only able to determine the intrafractional tumor motion,^6^, ^8^ but also to eliminate respiration motion artifacts.^9^, ^11^ Moreover, the size of PTVs of lung cancers can be reduced using the technique of 4D CT compared with the technique of 3D CT.^5^, ^12^, ^13^ This is particularly important in the radiotherapy of liver cancer, yet there are only a few reports on this field.^14^, ^16^

In this article, we initially defined a conventional PTV on axial 3D CT and an individual PTV on 4D CT. Then, two other individual PTVs were constructed, based on 3D CT combining with the data of individual tumor motion measured by 4D CT. The variations in target position, size, and inclusion relation between PTVs were compared. The purpose of this study is to analyze the characteristics of individual PTVs and provide evidences to select an appropriate PTV for the radiotherapy of liver cancer.

## II. MATERIALS AND METHODS

### A. Patient characteristics

Between September 2009 and November 2010, twenty patients with primary liver cancer underwent axial 3D CT and 4D CT simulation scanning for 3D CRT. All the patients received the therapy of segmental lipiodol‐transcatheter arterial chemoembolization (TACE) four weeks before CT scanning. The study was approved by the Institutional Review Board. Only 14 patients with homogneous accumulation or partial defect of lipiodol retention were included in target volume analysis. Twelve men and two women with a median age of 60 (range, 41–67) were included. There were 10 patients with tumors ≤5 mm in greatest dimension and two patients with tumors <5 mm, but <10 mm. The location of tumors in the liver is listed in Table 1.

**Table 1:** The location of tumors in the liver and the peak‐to‐peak displacement of center of mass (COM).

*Patient*	*Location*	*LR (mm)*	*AP (mm)*	*CC (mm)*	*3D (mm)*
1	LL, LHP	0.7	5.6	11.3	12.6
2	LL, LHP	1.7	5.7	13.4	14.7
3	RL, LHP	1.9	1.6	6.0	6.5
4	LL, LHP	0.9	3.0	5.5	6.3
5	RL, LHP	6.0	7.7	19.0	21.4
6	RL, LHP	2.0	1.3	2.4	3.4
7	RL, LHP	0.7	1.6	2.4	3.0
8	RL, LHP	1.5	6.2	10.2	12.0
9	LL, LHP	0.9	2.5	7.6	8.1
10	RL, UHP	0.8	3.1	8.9	9.5
11	RL, UHP	1.1	4.6	8.0	9.3
12	RL, UHP	1.5	2.8	15.0	15.3
13	LL, LHP	5.4	5.6	10.2	12.8
14	LL, UHP	0.6	2.9	8.8	9.3
Mean ‐	1.8	3.9	9.2	10.3
SD	‐	1.7	2.0	4.6	5.0

RL=right lobe of the liver, LL=left lobe, UHP = upper‐half part, LHP = lower‐half part, LR = left‐right direction, AP = anterior‐posterior, CC = cranial‐caudal, 3D=three‐dimensional motion vector.

### B. CT simulation and image acquisition

During the simulation, all patients were immobilized using vacuum bags in the supine position with the arms raised above the head. For each patient, an axial 3D CT scan of the upper abdomen region was performed, followed by a 4D CT scan during uncoached free breathing on Philips Brilliance Bores CT simulator (Philips Medical Systems, Highland Heights, OH). For 3D CT, each scan (360° rotation) took 1 s to acquire, followed by a 1.8 s dead time with a 2.4 cm coverage. The 3D CT scanning procedure takes about 30 s. During the 4D CT scanning, the respiratory signal recorded with real‐time positioning management (RPM) system (Varian Medical Systems, Palo Alto, CA) by tracking the trajectory of infrared markers placed on the upper abdomen was used for retrospective reconstructions. GE Advantage 4D software (GE Medical system, Milwaukee, WI) sorts the reconstructed 4D CT images into ten respiratory phases, with 0% corresponding to end‐inhalation, 50% corresponding to end‐exhalation. All the CT images were reconstructed used a thickness of 3 mm and then transferred to Eclipse treatment planning system (Eclipse 8.6, Varian Medical Systems, Palo Alto, CA).

### C. GTVs delineation

**Figure 1:**
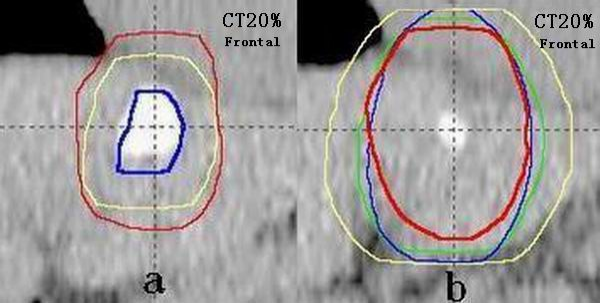
Example (Patient 1) of the different target volumes: (a) the ${\text{GTV}}_{3D}$ (blue line) and ${\text{CTV}}_{3D}$ (yellow line) derived from 3D CT, and ${\text{ITV}}_{4D}$ (red line) derived from 4D CT; (b) four PTVs based on 3D CT and 4D CT – ${\text{PTV}}_{4D}$ (red line), ${\text{PTV}}_{\text{conv}}$ (green line), ${\text{PTV}}_{\text{spec}}$ (blue line), and ${\text{PTV}}_{\text{vector}}$ (yellow line).

GTVs were manually delineated on the 10 phases of the 4D CT images by the same radiation oncologist using the same CT window setting (window width: 350 HU and window level: 40 HU). Because the 3D CT and 4D CT images for the same person were collected during the same imaging session, Eclipse considers the images as being registered with each other. 3D CT image was blended with 20% phase of 4D CT images, and then the GTV delineated on 3D CT would display on 20% phase of 4D CT images ((Fig. 1a).

### D. Tumor motion assessment

The coordinates in left–right (LR), anterior–posterior (AP), and cranial–caudal (CC) directions of the center of mass (COM) of GTVs in all respiratory phases were measured. The peak‐to‐peak displacement of COM in three directions was calculated based on the coordinates, which represents the tumor motion. The 3D motion vector (vector) of COM was calculated as follows:

(1) $$Vector=\sqrt{{LR}^{2}+{AP}^{2}+{CC}^{2}}$$

### E. PTVs definition and treatment planning

For the radiotherapy of liver cancer, a margin of 1.0 cm to 1.5 cm is adequate to account for microscopic extension (ME).^17^, ^18^ Therefore, we generated a clinical target volume (CTV) using a 1.0 cm expansion on the GTV ((Fig. 1a). For CTVs, the volume that is beyond liver tissues was not rectified, since the study is merely limited to volume comparison. Three internal target volumes (ITVs) were generated using different expansions on the CTV derived from 3D CT (${\text{CTV}}_{3D}$): i) a conventional ITV $\left({\mathrm{ITV}}_{\mathrm{conv}}\right)$ was produced by adding 10 mm in CC direction and 5 mm in LR and AP directions to ${\text{CTV}}_{3D}$; ii) a direction‐specific ITV $\left({\mathrm{ITV}}_{\mathrm{spec}}\right)$ was created using a specific margin in different directions, which was derived from the peak‐to‐peak displacement of COM in three directions measured by 4D CT; iii) ${\text{ITV}}_{\text{vector}}$ was produced by adding an isotropic margin derived from individual 3D motion vector of the tumor. ${\text{ITV}}_{4D}$ was defined on the fusion of CTVs on all phases of 4D CT ((Fig. 1a). Finally, ${\text{PTV}}_{\text{conv}}$, ${\text{PTV}}_{\text{spec}}$, ${\text{PTV}}_{\text{vector}}$, and ${\text{PTV}}_{4D}$ were generated by adding a 5 mm setup margin to ITVs ((Fig. 1b).

For all the patients, 3D CRT treatment planning was performed using the ${\text{PTV}}_{4D}$ and consisted of 3–5 coplanar or noncoplanar conformal beams. A daily dose of 2 Gy was administered at 5 fractions per week to deliver a total dose of 46 to 60 Gy.

### F. PTVs comparison

Position, volume, and degree of inclusion (DI) between ${\text{PTV}}_{\text{conv}}$, ${\text{PTV}}_{\text{spec}}$, ${\text{PTV}}_{\text{vector}}$, and ${\text{PTV}}_{4D}$ were compared, respectively. Positions of PTVs are represented by COM coordinates. The definition of DI of volume X included in volume Y (DI (X in Y)) is the ratio of the intersection between volume X and Y to volume X.^(19)^ The formula is as follows:

(2) $$DI\;(X\;in\;Y)=\frac{X\cap Y}{X}$$

We assumed volume Y was reference for the standard volume. If the treatment planning was based on volume X, there would be 1‐DI (X in Y) of volume X being unnecessary irradiated and 1‐DI (X in Y) of volume Y missing irradiation.

### G. Statistical analysis

Statistical analysis was performed using the SPSS software package (SPSS 16.0). The Wilcoxon test was used for comparison of position and volume of PTVs. A t‐test was used to test the variation of the tumor motion in different directions and degree of inclusion of PTVs. The variation of tumor motion for lesions in different locations of the liver was evaluated by the Wilcoxon test. The degree of associations between GTV motion vectors and continuous variables (e.g., volume and degree of inclusion) has been calculated by the Pearson test. Values of p < 0.05 were regarded as significant.

## III. RESULTS

The tumor motion amplitude for all patients is listed in Table 1. The tumor motion in the CC direction was greater than either LR (t=5.76, p < 0.001) or AP (t=3.49, p=0.001) direction. The mean tumor motion in the LR, AP and CC directions for tumors in the left lobe was 1.7±1.9  mm, 4.2±1.6  mm, and 9.5±2.8  mm, respectively, while for tumors in the right lobe, it was 1.3±0.5  mm, 3.3±1.9  mm, and 7.8±4.8  mm. The Wilcoxon test indicated no significant difference of tumor motion between two subgroups in the LR (p=0.331), AP (p=0.518), and CC (p=0.746) directions. The mean tumor motion vector in the CC direction was 10.2±3.2  mm for lesions in the upper‐half liver and 8.8±5.1  mm for lesions in the lower‐half liver. There is no significant difference between two groups (p=0.571).

${\text{CTV}}_{3D}$ centroid coordinates were used to represent the coordinates of PTVs derived from 3D CT due to isotropic expansions (at least in a single direction). Two related sample tests indicated no significant difference of centroid coordinates between ${\text{CTV}}_{3D}$ and ${\text{PTV}}_{4D}$ in the LR, AP, and CC directions (p=0.593, 0.421, and 0.889, respectively). Table 2 shows the shifts in centroid position between ${\text{CTV}}_{3D}$ and ${\text{PTV}}_{4D}$. The mean centroid shifts in the LR, AP, and CC directions between ${\text{CTV}}_{3D}$ and ${\text{PTV}}_{4D}$ were 0.2 mm, ‐0.4 mm, and 0.4 mm, respectively, which all approximated to zero.

**Table 2:** Centroid shifts of ${\text{CTV}}_{3D}$ and ${\text{PTV}}_{4D}$. The centroid of ${\text{CTV}}_{3D}$ is the centroid of PTVs derived from 3D CT.

*Patient*	${\text{CTV}}_{3D}$ ‐ ${\text{PTV}}_{4D}$			
	*LR (mm)*	*AP (mm)*	*CC (mm)*	*3D (mm)*
1	‐0.1	‐0.7	‐1.2	1.4
2	0.7	‐0.6	‐1.6	1.8
3	2.0	‐2.1	‐4.2	5.1
4	2.7	‐1.6	‐3.8	4.9
5	2.0	‐0.6	‐3.6	4.2
6	0.8	‐0.6	0.3	1.0
7	0.1	‐2.1	0.0	2.1
8	‐1.1	‐1.6	4.1	4.5
9	‐0.4	0.0	1.0	1.1
10	0.8	0.8	0.6	1.3
11	‐0.7	‐1.5	‐5.0	5.3
12	‐0.3	1.8	7.9	8.1
13	‐0.5	2.3	3.8	4.5
14	‐2.9	1.3	7.9	8.5
Mean	0.2	‐0.4	0.4	3.8
SD	1.4	1.4	4.2	2.5
Max (Abs)	2.9	2.3	7.9	8.5
Min (Abs)	0.1	0.0	0.0	1.0

Abs=the absolute.

PTVs size and the size ratio of PTVs derived from 3D CT (3D PTVs) to ${\text{PTV}}_{4D}$ are listed in Table 3. The mean volumetric reductions for ${\text{PTV}}_{4D}$ compared to ${\text{PTV}}_{\text{conv}}$, ${\text{PTV}}_{\text{spec}}$, and

**Table 3:** The absolute size of PTVs and the size ratio of PTVs derived from 3D CT and ${\text{PTV}}_{4D}$.

*Patient*	*Size of PTVs* ${\left(cm\right)}^{3}$				*The Size Ratio*		
	${\text{PTV}}_{4D}$	$PTV_{conv}$	$PTV_{spec}$	$PTV_{vector}$	$PTV_{conv}/PTV_{4D}$	$PTV_{spec}/PTV_{4D}$	$PTV_{vector}/PTV_{4D}$
1	105.74	145.14	140.63	248.13	1.37	1.33	2.35
2	65.00	101.26	111.03	207.83	1.56	1.71	3.20
3	73.53	106.52	76.40	113.18	1.45	1.04	1.54
4	102.23	192.49	142.05	178.59	1.88	1.39	1.75
5	703.35	796.97	1013.85	1588.27	1.13	1.44	2.26
6	334.55	529.99	383.92	425.33	1.58	1.15	1.27
7	326.07	512.96	371.65	414.00	1.57	1.14	1.27
8	106.69	162.78	156.54	255.48	1.53	1.47	2.39
9	71.60	120.19	96.14	139.73	1.68	1.34	1.95
10	88.27	143.27	116.25	191.31	1.62	1.32	2.17
11	62.35	152.39	138.45	185.92	2.44	2.22	2.98
12	146.33	199.92	201.95	401.40	1.37	1.38	2.74
13	349.59	397.25	407.02	592.41	1.14	1.16	1.69
14	338.82	467.14	410.12	555.52	1.38	1.21	1.64
Mean	205.29	284.16	269.00	392.65	1.55	1.38	2.09
SD	184.14	213.38	248.00	376.48	0.33	0.30	0.61
Median	106.22	177.64	149.30	251.81	1.54	1.34	2.06
Max	703.35	796.97	1013.85	1588.27	2.44	2.22	3.20
Min	62.35	101.26	76.40	113.18	1.13	1.04	1.27

${\text{PTV}}_{\text{vector}}$ were 33.18% ±12.39%, 24.95% ±13.01%, and 48.08% ±15.32%, respectively. The differences in size between ${\text{PTV}}_{4D}$ and ${\text{PTV}}_{\text{conv}}$, between ${\text{PTV}}_{4D}$ and ${\text{PTV}}_{\text{spec}}$, and between ${\text{PTV}}_{4D}$ and ${\text{PTV}}_{\text{vector}}$ were statistically significant (p=0.001, respectively). Comparing the size of PTVs from 3D CT, we found ${\text{PTV}}_{\text{conv}}$ size was larger than ${\text{PTV}}_{\text{spec}}$ (p=0.074), but smaller than ${\text{PTV}}_{\text{vector}}$ (p=0.041). ${\text{PTV}}_{\text{vector}}$ size was larger than ${\text{PTV}}_{\text{spec}}$ (p=0.001). The tumor motion vector showed a significant correlation to the size ratio of ${\text{PTV}}_{\text{vector}}$ to ${\text{PTV}}_{4D}$ (r=0.639, p=0.014), while it showed no significant correlation to the size ratios of ${\text{PTV}}_{\text{conv}}$ to ${\text{PTV}}_{4D}$ (r=‐0.446, p=0.110) and ${\text{PTV}}_{\text{spec}}$ to ${\text{PTV}}_{4D}$ (r=0.296, p=0.304).

**Figure 2:**
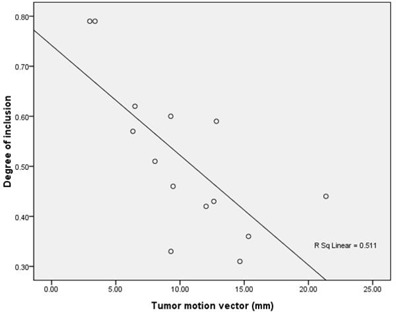
The correlation of DI of ${\text{PTV}}_{4D}$ in ${\text{PTV}}_{\text{vector}}$ to the tumor motion vector (r=‐0.715, p=0.004).

Table 4 shows the mutual degree of inclusion (DI) between ${\text{PTV}}_{4D}$ and ${\text{PTV}}_{\text{conv}}$, ${\text{PTV}}_{4D}$ and ${\text{PTV}}_{\text{spec}}$, and ${\text{PTV}}_{4D}$ and ${\text{PTV}}_{\text{vector}}$. The difference between DI of ${\text{PTV}}_{\text{conv}}$ in ${\text{PTV}}_{4D}$ and DI of ${\text{PTV}}_{\text{spec}}$ in ${\text{PTV}}_{4D}$ was not statistically significant (p=0.109). Both were greater than DI of ${\text{PTV}}_{\text{vector}}$ in ${\text{PTV}}_{4D}$ (p=0.012 and p < 0.001). The DI of ${\text{PTV}}_{4D}$ in ${\text{PTV}}_{\text{conv}}$, ${\text{PTV}}_{4D}$ in ${\text{PTV}}_{\text{spec}}$, and ${\text{PTV}}_{4D}$ in ${\text{PTV}}_{\text{vector}}$ approximated to 1, respectively, which showed no significant correlation to tumor motion vector (r=‐0.470, 0.259, and 0.244; p=0.090, 0.371, and 0.401, respectively). A significant inverse correlation was found for DI of ${\text{PTV}}_{\text{vector}}$ in ${\text{PTV}}_{4D}$ to tumor motion vector (r=‐0.714, p=0.004) (Fig. 2), while a significant positive correlation was found for DI of ${\text{PTV}}_{\text{conv}}$ in ${\text{PTV}}_{4D}$ to the motion vector (r=0.543, p=0.045). No significant correlation was found for DI of ${\text{PTV}}_{\text{spec}}$ in ${\text{PTV}}_{4D}$ to the motion vector (r=‐0.390, p=0.168).

**Table 4:** The mutual degree of inclusion (DI) between ${\text{PTV}}_{4D}$ and ${\text{PTV}}_{\text{conv}}$, ${\text{PTV}}_{4D}$ and PTV_spec_, and ${\text{PTV}}_{4D}$ and ${\text{PTV}}_{\text{vector}}$.

*Patient*	*Degree of Inclusion (DI) (%)*					
	$PTV_{conv}\;in\;PTV_{4D}$	$PTV_{4D}\;in\;PTV_{conv}$	$PTV_{spec}\;in\;PTV_{4D}$	$PTV_{4D}\;in\;PTV_{spec}$	$PTV_{vector}\;in\;PTV_{4D}$	$PTV_{4D}\;in\;PTV_{vector}$
1	72.47	99.48	74.85	99.55	42.61	100.00
2	64.04	99.77	58.38	99.72	31.28	100.00
3	66.43	96.23	80.79	83.94	61.96	95.38
4	52.98	99.76	70.31	97.70	57.24	100.00
5	85.86	97.29	69.20	99.75	44.28	100.00
6	63.11	99.98	86.31	99.05	78.56	99.87
7	63.56	99.99	85.83	97.83	78.50	99.67
8	63.45	96.80	65.84	96.60	41.76	100.00
9	59.55	99.96	74.37	99.86	51.24	100.00
10	61.59	99.97	75.78	99.81	46.14	100.00
11	40.30	98.51	44.34	98.46	33.04	98.52
12	68.12	93.07	70.33	97.06	36.45	100.00
13	85.02	96.61	83.66	97.40	59.01	99.99
14	70.24	96.85	78.17	94.62	59.77	98.00
Mean	65.48	98.16	72.73	97.24	51.56	99.39
SD	11.54	2.08	11.33	4.12	15.09	1.31
Median	63.80	98.99	74.61	98.15	48.69	100.00
Max	85.86	99.99	86.31	99.86	78.56	100.00
Min	40.30	93.07	44.34	83.94	31.28	95.38
*t*		‐10.443		‐7.585		‐11.674
*p*		0.000		0.000		0.000

## IV. DISCUSSION

Liver is subject to large movement during normal breathing.^(20)^ Precisely accounting for respiration‐induced liver tumor motion is able to increase the accuracy of PTV determination. We examined the characteristics of liver tumor motion using the 4D CT. Our data suggested that the mean tumor motion in the CC direction was 9.2±4.6  mm, but it was not beyond 5 mm in the LR or AP direction. The tumor motion vector was 10.3±5.0  mm. These data matched results reported in other literature.^14^, ^21^ Xi et al.^(14)^ assessed the liver tumor motion of 10 patients using 4D CT, and showed the mean tumor motion was 3.2±1.1  mm, 3.5±1.1  mm, and 11.0±5.4  mm in the LR, AP, and CC directions, respectively. Case et al.^(21)^ reported the 3D vector liver motion amplitude measured on the planning 4D CTs was 10.1 (range, 2.4–19.4) mm. The liver tumor motion examined using high‐speed MRI^(22)^ or respiratory correlated cone beam CT (rcCBCT)^(21)^ was similar to the results accessed using 4D CT. All the data indicated that the tumor motion in CC direction was significantly greater than that in LR or AP direction. It is necessary to use an anisotropic margin in different directions to account for tumor motion.

The tumor motion amplitude may be different for tumors in different locations of the liver. Kitamura et al.^(23)^ reported that the tumor motion of the right liver lobe was significantly larger than that of the left lobe in the LR and AP directions (p < 0.01). However, in our study no obvious variations in tumor motion were found for tumors in the right lobe and the left lobe in the LR (p=0.331), AP (p=0.518) and CC (p=0.746). We currently cannot be sure which study was correct due to the limited cases for the two studies. In addition, we found the tumor motion of the upper‐half liver (mean 10.2 mm) was larger than that of the lower‐half liver (mean 8.8 mm) (p=0.571). Kim et al.^(24)^ observed the mean CC movements of the hepatic dome and lower tip in the supine position were 15.0 mm and 12.8 mm. It seemed that CC location of the liver tumor only had a small effect on tumor motion, while CC location of the lung tumor emerged as the most important factor correlated with tumor motion.^(6)^ We should pay attention to the difference between liver and lung tumors.

${\text{PTV}}_{4D}$ centroid position could represent the average target position due to 4D CT including all phases throughout the breathing cycle. However, axial 3D CT scanning could only provide a snapshot of anatomy, so we speculated ${\text{CTV}}_{3D}$ centroid merely represented a random target position. Our study confirmed this speculation. The average centroid shifts between ${\text{CTV}}_{3D}$ and ${\text{PTV}}_{4D}$ in the LR, AP, and CC directions were close to zero. That is to say, ${\text{CTV}}_{3D}$ centroids randomly scatter around the center of ${\text{PTV}}_{4D}$. Our study in liver cancer was in agreement with the study in lung caner reported by other authors.^5^, ^25^ Therefore, the margin accounting for tumor motion should be isotropic in a single direction for 3D CT treatment planning.

We further analyzed the variation in volume between 3D PTVs and ${\text{PTV}}_{4D}$ to reveal the impact of different CTV‐to‐ITV expansions on determining the PTV. Our study showed the mean volumetric reductions for ${\text{PTV}}_{4D}$ compared to ${\text{PTV}}_{\text{conv}}$, ${\text{PTV}}_{\text{spec}}$, and ${\text{PTV}}_{\text{vector}}$ were 33.18%, 24.95%, and 48.08%, respectively. Recent data by other authors^14^, ^19^ support our observation. Xi et al.^(14)^ compared the difference in size between ${\text{PTV}}_{4D}$ and ${\text{PTV}}_{3D}$ derived from a single CTV (20% phase) using conventional margins. They found ${\text{PTV}}_{4D}$ has a 19% decrease on average than that of ${\text{PTV}}_{3D}$. Hof et al.^(19)^ reported a mean PTV reduction of 31% by 4D CT‐based PTV compared to a fast CT‐based PTV using individual margins. Additionally, we found ${\text{PTV}}_{\text{vector}}$ size was significantly larger than ${\text{PTV}}_{\text{conv}}$ (p=0.041) or ${\text{PTV}}_{\text{spec}}$ (p=0.001), while no obvious difference between ${\text{PTV}}_{\text{conv}}$ and ${\text{PTV}}_{\text{spec}}$ was found (p=0.158). So we know the use of an isotropic margin derived from individual 3D motion vector on 3D CT would increase the PTV size significantly, while the use of a direction‐specific margin derived from individual tumor motion could not reduce the PTV size, compared to the conventional PTV.

The overlapping relationship between 3D PTVs and ${\text{PTV}}_{4D}$ cannot be revealed if we just compare volumetric difference of PTVs. So, we introduced the concept of degree of inclusion (DI). Our data showed that the mean DIs of ${\text{PTV}}_{4D}$ in ${\text{PTV}}_{\text{conv}}$, ${\text{PTV}}_{4D}$ in ${\text{PTV}}_{\text{spec}}$, ${\text{PTV}}_{4D}$ in ${\text{PTV}}_{\text{vector}}$ were close to 100%, while the minimum DIs were 93.07%, 83.94%, and 95.38%, respectively. These results indicated the use of 3D PTVs in treatment planning would not result in a serious geometric miss, compared the use of ${\text{PTV}}_{4D}$. Therefore, the key to our study was the volume of normal tissue unnecessarily irradiated. Unfortunately, there would be 34.52%, 27.27%, or 48.44% of normal tissue unnecessarily irradiated on average if we use ${\text{PTV}}_{\text{conv}}$, ${\text{PTV}}_{\text{spec}}$, and ${\text{PTV}}_{\text{vector}}$ in the treatment planning. Further research suggested that the difference between DIs of ${\text{PTV}}_{\text{conv}}$ in ${\text{PTV}}_{4D}$ and ${\text{PTV}}_{\text{spec}}$ in ${\text{PTV}}_{4D}$ was not significant (p=0.109). The use of ${\text{PTV}}_{\text{spec}}$ in treatment planning did not show a greater advantage on reducing the normal tissue unnecessarily irradiated than the use of ${\text{PTV}}_{\text{conv}}$.

When evaluating the correlation of DIs and 3D motion vector, we found DI of ${\text{PTV}}_{\text{vector}}$ in ${\text{PTV}}_{4D}$ became worse as the motion vector increased (r=‐0.714, p=0.004), while DI of ${\text{PTV}}_{4D}$ in ${\text{PTV}}_{\text{vector}}$ showed no apparent change (p=0.401). In addition, we found DI of ${\text{PTV}}_{\text{conv}}$ in ${\text{PTV}}_{4D}$ increased as the motion vector increased (r=0.543, p=0.045), but DI of ${\text{PTV}}_{4D}$ in ${\text{PTV}}_{\text{conv}}$ demonstrated a trend of decease (r=‐0.470, p=0.090). In other words, the risk of missing target would increase as the tumor motion increases, if we use ${\text{PTV}}_{\text{conv}}$ in the treatment.

In this study, we generated the CTV using a 1.0 cm expansion on the GTV, and then the PTVs were defined for conventionally fractionated 3D CRT in patients with HCC. However, the GTV was usually considered to be identical to the CTV in the stereotactic body radiation therapy (SBRT).^26^, ^27^ The patients tolerated high‐radiation dose treatments well, and a high tumor control rate could be achieved in the SBRT of liver cancer.^26^, ^27^ The use of the individual PTV based on 4D CT can significantly reduce the PTV size, but it can't eliminate the impact of the generation of the CTV on increasing the size. Therefore, it may potentially decrease patient tolerance, if the PTVs accounting for ME were used in the SBRT of liver cancer.

One limitation of this study is that ${\text{PTV}}_{4D}$ was regarded as the reference for standard volume. Respiratory variations^(28)^ and the delineation error^(29)^ may reduce the accuracy of ${\text{PTV}}_{4D}$. Moreover, 4D CT may not accurately reflect the range of tumor motion due to the irregular tumor motion in amplitude and periodicity, and the intraphase residual motion exist in 4D CT. ^30^, ^31^ Another margin should be used to compensate the uncertainty. The inaccurate ${\text{PTV}}_{4D}$ would have adverse impact on the study. In fact, the edge of lesions may be blurring on the 4D CT images for patients who did not undergo TACE, which may limit the use of 4D CT in the radiotherapy of liver cancer.

In addition, we only used CT images for GTV delineation in this study. However, the use of CT images alone for GTV delineation may miss potential tumor cell congregations.^(32)^ The target volumes of liver metastases delineated on contrast‐enhanced CT was significantly smaller than that on MRI.^(32)^ Méndez Romero et al.^(33)^ indicated a good correlation between the colorectal liver metastases dimensions measured by MRI and the macroscopic pathology, suggesting MRI can be used for accurate target delineation. The use of MRI fused with CT to contour the GTV is recommended.

## V. CONCLUSIONS

We have analyzed characteristics of the liver tumor motion using 4D CT and found the location of lesions in the liver had no obvious impact on tumor motion. The position of targets derived from axial 3D CT images scatter around the center of 4D targets. It is necessary to expand an isotropic margin in a single direction to account for tumor motion for 3D CT treatment planning. The size of individual PTV derived from 4D CT is significantly less than that of PTVs derived from 3D CT. The 3D CT‐based PTVs provide a good coverage of the 4D CT‐based PTV, but encompass relatively large normal tissues. Compared to the conventional PTV, the use of 3D CT‐based PTVs with individual margins cannot significantly reduce normal tissues being unnecessarily irradiated, but may contribute to reducing the risk of missing targets for tumors with large motion.
